# In silico Identification of Natural Compounds as Potential TrkA Inhibitors for Anticancer Drug Development

**DOI:** 10.5812/ijpr-166946

**Published:** 2026-05-25

**Authors:** Mujtaba Fadhil, Dhurgham Al-Fahad, Faizul Azam, Asadollah Asadi

**Affiliations:** 1 Department of Biology, Faculty of Science, University of Mohaghegh Ardabili, Ardabil, Iran; 2 Department of Pathological Analysis, College of Science, University of Thi-Qar, Nasiriyah, Iraq; 3 Department of Medicinal Chemistry and Pharmacognosy, College of Pharmacy, Qassim University, Buraydah, Saudi Arabia

**Keywords:** TrkA, Cancer Cell Migration, Docking, Molecular Dynamics Simulations, MM-GBSA

## Abstract

**Background:**

Tropomyosin receptor tyrosine kinase A (TrkA) is essential for cancer cell migration and for stabilizing focal adhesions required for attachment to the extracellular matrix. However, resistance to inhibitors targeting the ATP-binding pocket of the TrkA kinase domain complicates treatment. These inhibitors block ATP binding, which is required for TrkA activation, thereby disrupting downstream signaling pathways involved in cell proliferation and survival in cancers characterized by TrkA overexpression or mutation.

**Objectives:**

This study aimed to identify natural compounds as potential TrkA inhibitors for anticancer drug development. The identified compounds exhibited higher binding affinity and greater stability than the control compounds.

**Methods:**

Virtual screening, molecular dynamics simulations, MM-GBSA free energy calculations, and principal component analysis were performed using the Schrödinger Desmond software to screen a comprehensive library from the NPACT and PhytoHub databases for potential novel TrkA inhibitors.

**Results:**

Two promising inhibitors derived from natural compounds, PHUB000399 and NPACT01417, were identified. Toxicity assessments indicated that both compounds had lower toxicity levels (class 5) than Entrectinib, the reference inhibitor (class 4); the molecular weights of PHUB000399, NPACT01417, and Entrectinib were 275.25, 316.35, and 560.64 g/mol, respectively. Docking studies showed that PHUB000399 and NPACT01417 had superior binding affinities, with grid scores of -13.239 and -13.103, respectively, compared with the co-crystal ligand (-12.567) and Entrectinib (-10.996). Molecular dynamics simulations indicated that the TrkA-PHUB000399 and TrkA-NPACT01417 complexes were more stable than the TrkA-Entrectinib complex. The calculated binding free energies for TrkA-PHUB000399 and TrkA-NPACT01417 were -87.74 and -86.09 kcal/mol, respectively, exceeding those of the co-crystal ligand (-84.14 kcal/mol) and Entrectinib (-73.03 kcal/mol).

**Conclusions:**

These findings suggest that NPACT01417 and PHUB000399 are promising candidates for targeting cancer cell migration, with binding affinities comparable to those of established inhibitors. This study provides valuable insights for the development of effective anticancer therapies.

## 1. Background

Tropomyosin receptor tyrosine kinases (TRKs) are a unique family of transmembrane glycoproteins that are essential for diverse neuronal functions (1). They comprise 3 subtypes, TRKA, TRKB, and TRKC, which are located primarily in the nervous system. The genes encoding these TRK enzymes, neurotrophic tyrosine receptor kinase (NTRK) genes, can undergo fusion mutations (2). These alterations cause structural changes in the extracellular domain of TRK enzymes, resulting in constitutive activation associated with various cancers, including rare cancers such as thyroid cancer and infantile fibrosarcoma, as well as more common cancers such as lung and colon cancers (3). Addressing these oncogenic mutations requires selective small-molecule TRK inhibitors, which are increasingly used in targeted cancer therapies (4).

Each subtype is activated by a specific neurotrophin. For example, TRKA is activated by nerve growth factor, which initiates MAPK signaling pathways, influences cell growth, and plays a significant role in pain and inflammation. Similarly, TRKB, which is activated by neurotrophin-4 and brain-derived neurotrophic factor, engages pathways such as RAS/ERK and PI3K, which are essential for neural cell plasticity and metabolism. TRKC is activated by neurotrophin-3 and primarily stimulates the PI3K/AKT and MAP4K4 pathways (1, 5). The MAP4K4 pathway has an important role in cancer cell migration by activating and stabilizing focal adhesion proteins such as zyxin and vinculin, which are crucial for cancer cell movement through adhesion and detachment processes (6-8).

Among the TRK subtypes, tropomyosin receptor tyrosine kinase A (TrkA) is particularly important because it mediates neurotrophic signaling and influences neuronal survival, differentiation, and function. Dysregulation of TrkA signaling is linked to several neurological disorders, including neurodegenerative diseases, chronic pain, and cancer. Therefore, targeting TrkA is a promising therapeutic strategy. However, traditional drug development approaches have struggled to identify effective inhibitors that modulate TrkA activity without adverse effects. TRK inhibitors can be classified into 3 types according to their binding sites. Type I and type II inhibitors bind to the adenosine triphosphate (ATP)-binding site, with type I inhibitors acting on the active form of the enzyme (DFG-in) and type II inhibitors interacting with the inactive form (DFG-out). In contrast, type III inhibitors target an allosteric site distinct from the ATP-binding site. The chemical structure of type I TRK inhibitors typically consists of a head, a core ring, and a tail, with specific residues in the ATP-binding site playing crucial roles in the interaction (1, 5).

Two type I TRK inhibitors, Larotrectinib (Vitrakvi®) and Entrectinib (Rozlytrek®), have received FDA approval for treating NTRK fusion-positive cancers and have demonstrated significant clinical efficacy. Despite their success, resistance driven by mutations in TRK enzymes remains challenging. These mutations can occur at critical sites, hinder inhibitor binding, and require innovative strategies to overcome acquired resistance. Approaches such as sequential treatment with different inhibitors and the development of second-generation compounds are being explored to address this issue. The development of selective TRKA inhibitors is also complicated by the similarity among TRK subtypes in their ATP-binding sites. A promising strategy involves targeting the allosteric pocket, which differs among the subtypes and can allow greater selectivity of type III inhibitors for TRKA. This selectivity may help minimize on-target adverse effects, particularly in the central nervous system, where TRKA is crucial for regulating pain and inflammation (9).

Multiple strategies have been applied to address acquired resistance. Clinically, sequential treatment with Larotrectinib and Entrectinib in patients with NTRK fusion-positive sarcoma has been shown to be effective. In 1 case, a patient was treated with Entrectinib for 4 months, followed by Larotrectinib after resistance to the first drug developed. In addition, the development of second-generation type I inhibitors and the use of type II or type III TRK inhibitors can help overcome acquired resistance (10). Developing selective TRKA inhibitors remains difficult because of the similarities among the 3 subtypes in the ATP-binding site. A more effective strategy is to target the allosteric pocket, which varies in size among TRK subtypes. Therefore, most type III inhibitors are selective for the TRKA subtype. Selective TRK inhibitors can reduce the on-target adverse effects observed with type I inhibitors, particularly in the central nervous system. In addition, TRKA has a crucial role in regulating pain and inflammation (11, 12).

Phytochemicals show significant therapeutic potential by modulating biological pathways associated with TrkA-related conditions, such as neurodegenerative diseases and cancer. Their natural origins often suggest better safety profiles, making them promising candidates for drug development (13). NPACT01417 originates from Larrea tridentata, which belongs to the family Zygophyllaceae (14), whereas PHUB000399 is a naturally occurring trihydroxyflavanone, a bioactive flavonoid compound found in plants such as Dalbergia odorifera, which belongs to the family Fabaceae (15).

**Figure 1.**
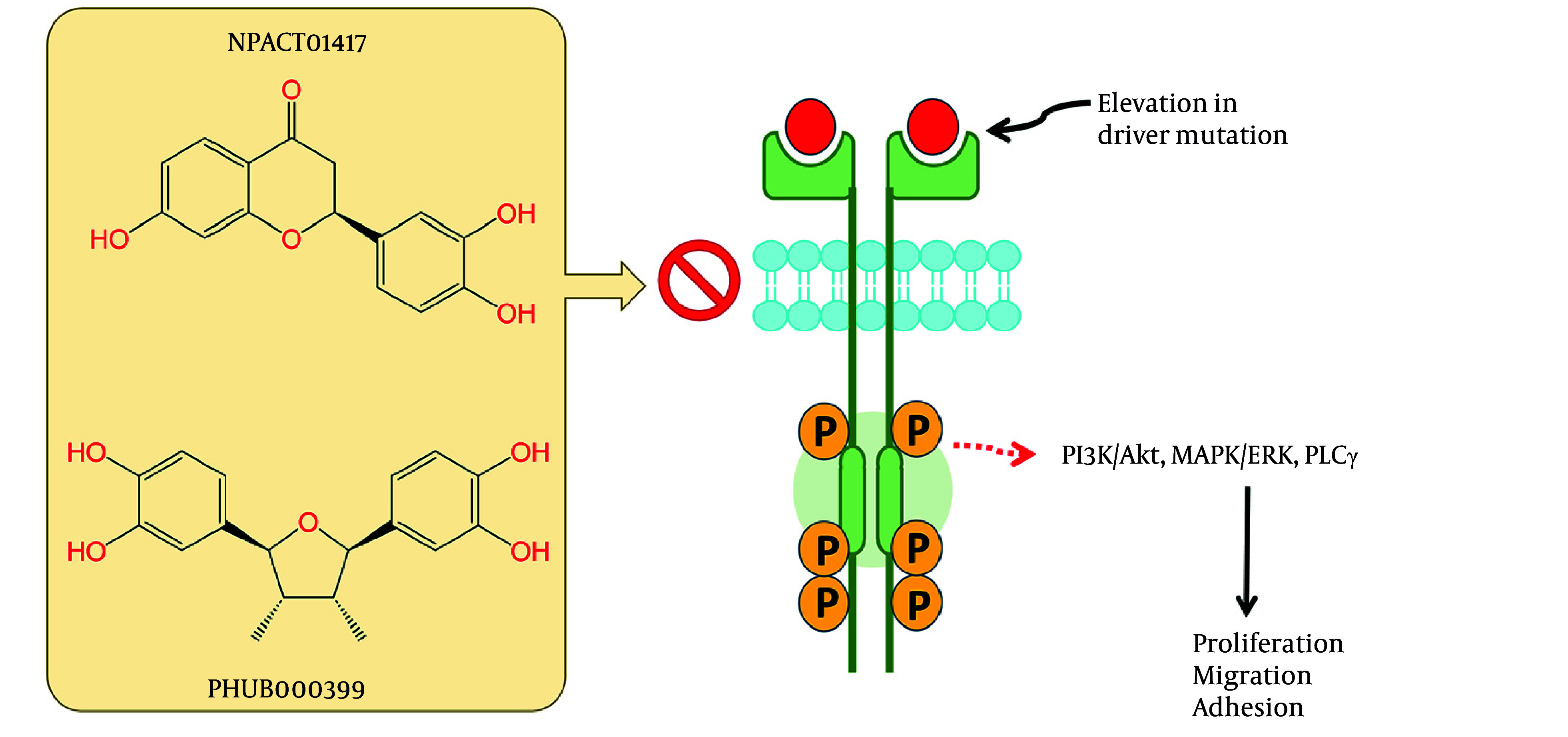
The diagram illustrates the signaling pathways activated by TrkA kinase and highlights the potential inhibitors NPACT01417 and PHUB000399 that target TrkA. TrkA activates key pathways, including PI3K/AKT, which is involved in cell proliferation and survival; MAPK/ERK, which is critical for cell migration and differentiation; and PLC, which contributes to cell adhesion and other essential functions. However, mutations in the TrkA gene can lead to resistance to specific inhibitors, complicating treatment strategies. NPACT01417 and PHUB000399 are presented as promising inhibitors that may effectively target and inhibit TrkA and potentially overcome this resistance.

In recent years, computational methods, particularly in silico studies, have emerged as powerful tools for predicting potential inhibitors and optimizing drug candidates (16, 17). These techniques allow researchers to efficiently explore vast chemical spaces, identify potential binding interactions, and evaluate the pharmacokinetic properties of candidate molecules before experimental validation (18, 19). This study used in silico techniques, including molecular docking, virtual screening, and molecular dynamics simulations, to identify and characterize potential inhibitors of TrkA. By identifying novel compounds that selectively inhibit TrkA signaling (Figure 1), this study may contribute to the development of targeted therapies for TrkA-related disorders and improve treatment efficacy while minimizing adverse effects.

## 2. Objectives

This study aimed to identify natural compounds as potential TrkA inhibitors for anticancer drug development. Specifically, virtual screening, molecular docking, molecular dynamics simulations, MM-GBSA binding free energy calculations, and essential dynamics analyses were used to evaluate natural compounds from the NPACT and PhytoHub databases and compare the most promising hits with the co-crystal ligand and Entrectinib.

## 3. Methods

### 3.1. Protein Preparation and Preparation of the Chemical Database

The crystal structure of human TrkA kinase bound to its inhibitor (PDB accession ID 7XBI) was retrieved from the Protein Data Bank (20). Using the Protein Preparation Wizard, missing hydrogen atoms were added, and water molecules trapped during X-ray crystallography were removed. Bond orders were assigned, and terminal residues were capped. Disulfide bonds were formed between proximal sulfur atoms, whereas other parameters were left unchanged. The final refinement step involved comprehensive energy minimization using the OPLS3 force field, with the RMSD of heavy atoms set to 0.3 Å (20, 21). A Glide grid file was then generated using the receptor grid generation panel, in which the co-crystallized ligand, 4-[[2-fluoro-5-(trifluoromethyl)phenyl]carbamoylamino]-N-[3-(1-methylpyrazol-4-yl)-1H-indazol-5-yl]-2-(trifluoromethyl)benzamide, located in the active site, was selected. For virtual screening, 2 large chemical libraries, comprising 1574 compounds from NPACT (http://crdd.osdd.net/raghava/npact/) and 800 phyto-compounds from the PhytoHub database (https://phytohub.eu/), were downloaded (22). The LigPrep module of the Schrödinger suite was used to convert the downloaded phyto-compounds from SMILES format. During ligand preparation, protonation states were adjusted to pH 7.4 ± 1.0, hydrogen atoms were added, and ligand energies were minimized using the OPLS3 force field, with all other settings kept at default.

### 3.2. Hierarchical Virtual Screening

A virtual screening approach was used to evaluate phyto-compounds from the NPACT and PhytoHub databases. The screening protocol consisted of 3 docking levels: high-throughput virtual screening (HTVS), standard precision (SP), and extra precision (XP). The workflow was performed using the Virtual Screening Workflow module in the Schrödinger molecular modeling suite. This protocol involves 3 successive docking stages, with the output of each stage serving as the input for the subsequent stage (23 - 25). At each docking phase, the user can define the percentage of docked compounds to retain. Initially, all phyto-compounds were docked to the TrkA receptor using HTVS, generating a single conformation for each molecule. The top 50% of the best-performing compounds were then carried forward to SP docking. After SP docking, the top 50% of compounds were again selected for XP docking. Finally, XP descriptor data were recorded to support further analysis of binding interactions.

These phyto-compounds underwent stepwise drug-likeness and developability filtering using calculated physicochemical descriptors and substructure alerts. Initially, Lipinski rule-of-5 parameters, including molecular weight, lipophilicity, hydrogen-bond donors, and hydrogen-bond acceptors, were calculated, and only compounds with no rule-of-5 violations were selected for subsequent analysis (26). Compounds meeting the rule-of-5 criteria were then assessed according to the Veber criteria for oral bioavailability, using thresholds related to molecular flexibility and polarity, specifically the number of rotatable bonds and polar surface area/hydrogen-bonding capacity (27). To evaluate practical tractability, synthetic accessibility was assessed using the fragment-contribution/complexity-based synthetic accessibility scoring scheme on a scale of 1 to 10, and compounds with a synthetic accessibility score ≤ 6.0 were accepted (28). Overall drug-likeness was summarized using the quantitative estimate of drug-likeness, and molecules with a quantitative estimate of drug-likeness ≥ 0.5 were selected (29). Shortlisted candidates were screened using PAINS substructure filters to eliminate compounds with motifs associated with common assay interference; only PAINS-negative molecules were advanced (30).

### 3.3. Molecular Dynamics Simulation

To investigate the stability and molecular interactions of the proposed phyto-compounds, molecular dynamics simulations were performed using Schrödinger Desmond software. The best-docked conformations of phyto-compounds showing strong interactions with the TrkA protein were selected for simulations. The systems were solvated using a simple point charge water model, and electroneutrality was established by adding sodium and chloride ions. Using the Desmond System Builder panel, a 0.15 M NaCl concentration was set to mimic physiological conditions (31, 32). The system, prepared with the OPLS3e force field, underwent energy minimization using the steepest descent method to resolve steric clashes. The system was then equilibrated under an NPT ensemble (isothermal-isobaric) for 100 ps, with temperature maintained at 300 K using a Nose-Hoover chain thermostat and pressure maintained at 1.0315 bar using the Martyna-Tobias-Klein barostat (33). The molecular dynamics simulation was then extended for 100 ns, with trajectory snapshots saved every 100 ps. Ligand stability and binding orientation were analyzed using the Desmond Simulation Interaction Diagram by examining 1000 molecular dynamics trajectory frames. MM-GBSA binding free energy calculations were performed on 1000 frames from each molecular dynamics trajectory.

## 4. Results and Discussions

### 4.1. Hierarchical Virtual Screening

**Figure 2.**
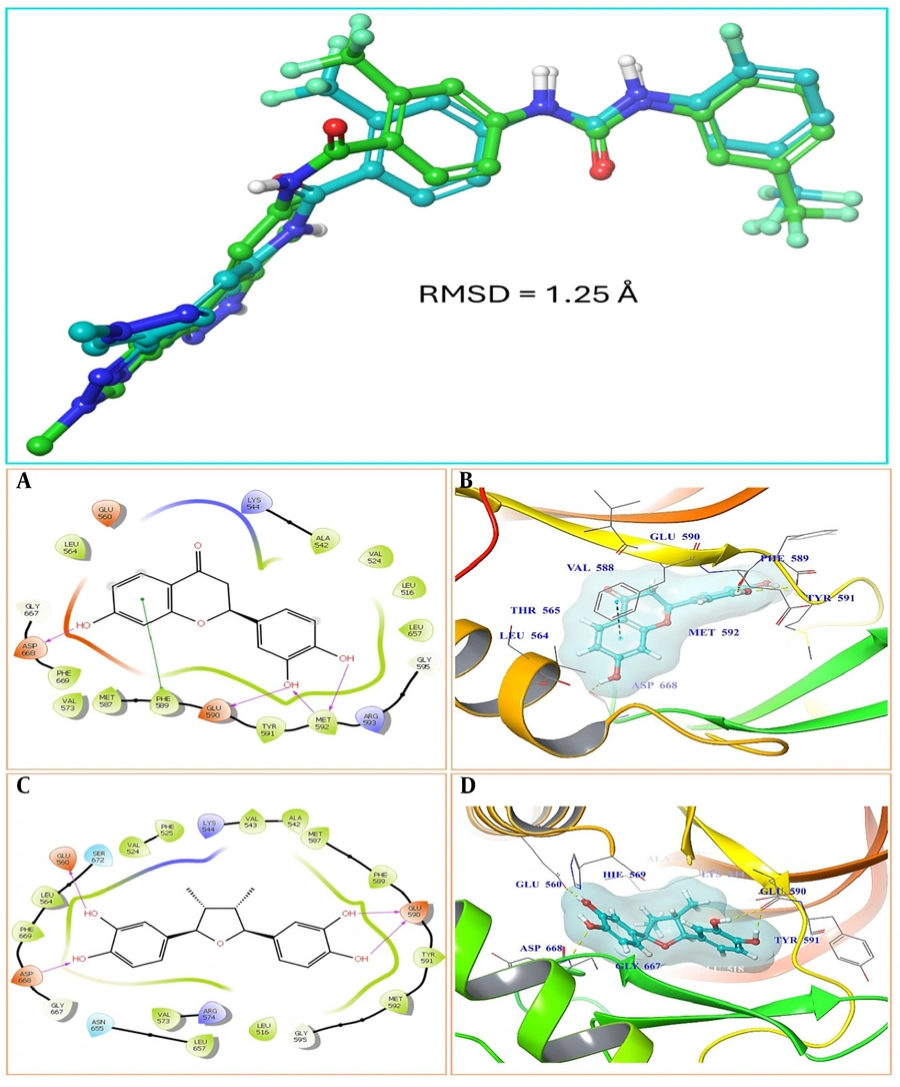
Docking validation by overlapping. A and B, Docked poses of the co-crystal ligand; C and D, specific inhibitor Entrectinib on TrkA kinase.

A hierarchical virtual screening approach was applied to a dataset of 1574 compounds from the NPACT database and 800 compounds from the PhytoHub database, targeting TrkA kinase (PDB ID: 7XBI). In the initial HTVS phase, 1169 compounds were selected for SP docking. The docking scores of the co-crystallized ligand and the control drug Entrectinib were used as reference points to identify the most promising compounds. If the RMSD between the co-crystal pose and docked pose is below 2 Å, the docking protocol is considered validated (34). SP docking showed that the docked pose of the co-crystal ligand closely replicated the co-crystal pose, with an RMSD of 1.254 Å (Figure 2). The docking scores for the co-crystallized ligand and Entrectinib were -12.567 and -10.996 kcal/mol, respectively. In addition, the co-crystal ligand originally exhibited key hydrogen-bond interactions with Glu560, Met592, Glu560, and Asp668 (Figure 2). The standard drug Entrectinib formed hydrogen bonds with Met592 and Glu590 residues (Figure 2). SP docking of the 1169 compounds identified 585 compounds, which were further subjected to XP docking. In this final XP docking stage, 291 compounds were obtained, with docking scores ranging from -17.827 to -10.656 kcal/mol. Compounds with XP docking scores lower than -12 kcal/mol were considered for further analysis. Subsequently, these compounds were filtered using the Lipinski rule of 5 to assess drug-likeness (26). Twenty phyto-compounds with no Lipinski rule-of-5 violations were identified. These 20 phytocompounds were further subjected to developability assessment.

All shortlisted molecules complied with the Veber filter and met established criteria for synthetic accessibility (synthetic accessibility score ≤ 6.0) and quantitative drug-likeness (quantitative estimate of drug-likeness ≥ 0.5). In addition, none triggered PAINS structural alerts. Collectively, these outcomes support selection of a chemically tractable, drug-like subset for subsequent analysis. Lipinski- and Veber-type rules serve as early heuristics to identify compounds with favorable oral absorption properties, whereas the synthetic accessibility score provides a rapid assessment of synthetic feasibility, and the quantitative estimate of drug-likeness consolidates multiple physicochemical characteristics into a single desirability metric. PAINS filtering reduces the likelihood of selecting frequent hitters or motifs that interfere with assays. However, these filters should be regarded as risk-reduction tools rather than conclusive proof of distinct, clean bioactivity.

**Figure 3.**
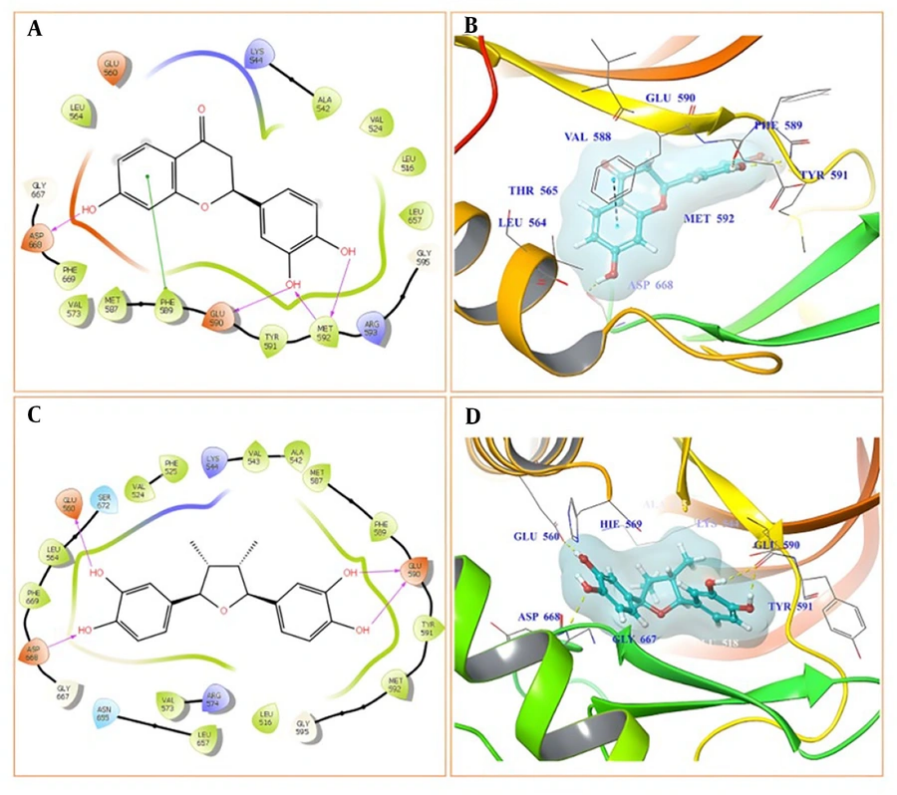
Docked poses of potential inhibitors. A, PHUB000399; B, NPACT01417 on TrkA kinase.

Binding interactions were further analyzed based on the reported interactions of the co-crystal ligand and control drug. Key residues involved in TrkA binding, including Asp668 in the DFG motif, Met592, and Glu590, were determined to be crucial for ligand binding. Two compounds, PHUB000399 (docking score = -13.239 kcal/mol; Figure 3A) and NPACT01417 (docking score = -13.103 kcal/mol; Figure 3B), showed binding interactions with these crucial residues similar to those of the co-crystal ligand and control drug (Table 1). These interactions suggest that PHUB000399 and NPACT01417 may act as potential TrkA inhibitors and warrant further investigation. The docking poses of the co-crystal ligand and Entrectinib are shown in Figure 2, whereas the poses of the top-hit compounds, PHUB000399 and NPACT01417, are shown in Figure 3.

**Table 1.** Docking Scores and MM-GBSA Binding Energies of the Selected TrkA Kinase Inhibitors

Compound	Docking Score (kcal/mol)	MM-GBSA Binding Energy (kcal/mol)	Hydrogen Bonds	Key Residues Interacted	Hydrophobic Interactions
**PHUB000399**	-13.239	-87.74	3	Glu590, Met592, Asp668	π-alkyl, π-π stacking, π-sulfur
**NPACT01417**	-13.103	-86.09	2	Glu590, Asp668	π-alkyl, hydrophobic
**Co-crystal ligand**	-12.567	-84.14	2	Glu590, Asp668	Hydrophobic
**Entrectinib**	-10.996	-73.03	1	Thr152	Hydrophobic

### 4.2. Molecular Dynamics Simulations

**Figure 4.**
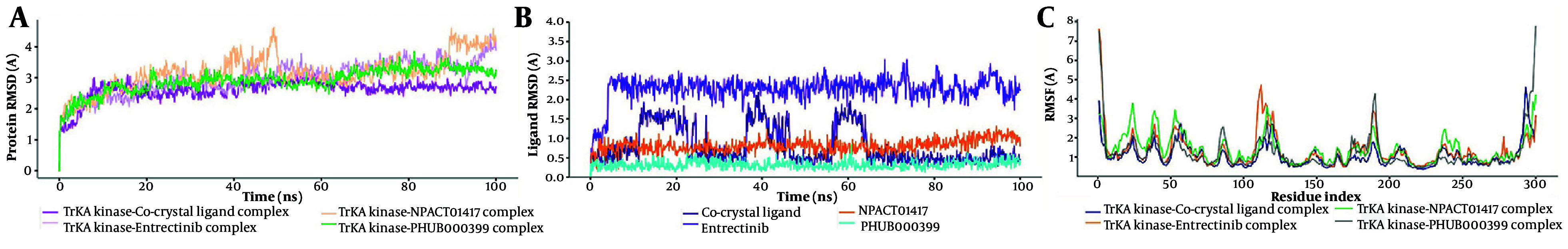
Molecular dynamics simulation analysis results. A, RMSD in TrkA backbone atoms; B, RMSD in respective ligands; C, RMSF.

Molecular dynamics simulation studies were performed to investigate the stability of protein-ligand interactions. Complexes of the TrkA receptor with the co-crystal ligand, the control drug Entrectinib, PHUB000399, and NPACT01417 were selected for molecular dynamics simulation studies. The results of RMSD and RMSF analyses for all systems are summarized in Figure 4.

The RMSD of protein Cα atoms and ligand atoms is considered stable if it is less than 2 Å (35). The RMSD of the Cα atoms of the TrkA receptor was slightly higher, ranging from 2.4 to 2.8 Å. The complex equilibrated at 2.8 Å and exhibited wave-like behavior until 60 ns. The complex showed a higher RMSD of approximately 3.0 Å at 60 ns and then stabilized at a uniform point until the end of the simulation. The RMSD of the co-crystal ligand deviated substantially for approximately 65 ns of the simulation period and then stabilized, with an average RMSD of approximately 1.6 Å. During this period, the ligand underwent significant perturbation in the binding cavity. The RMSF for residues in the loop regions, encompassing residues 50 to 65 and 105 to 140, was markedly higher, reaching up to 2.4 Å. Residues in loop regions are flexible because of their unstructured nature (36). Residues involved in interactions with the co-crystal ligand showed reasonably low and stable RMSF values below 1.2 Å (Figure 4A).

The RMSD of the Cα atoms of the TrkA receptor in complex with Entrectinib was significantly higher, with an average of approximately 3.0 Å (Figure 4A). The RMSD of Entrectinib atoms remained reasonably stable from 20 to 60 ns of the simulation, with an average of approximately 2.5 Å. However, larger deviations were observed thereafter until the end of the simulation period. This indicates that the protein RMSD increased gradually but stabilized between 2 and 3 Å, suggesting relatively constrained conformational variations. The results suggest that the initial TrkA conformation underwent substantial conformational changes during the simulation. The ligand RMSD showed increased variability, with deviations up to approximately 4 Å, particularly between 70 and 85 ns, suggesting an inherently flexible and dynamic binding pocket. In contrast, the consistency in protein RMSD relative to ligand RMSD indicates that the protein structure remained stable while the ligand explored multiple binding conformations. Major conformational changes were also evident from the significantly higher RMSF of residues in the loop regions encompassing residues 100 to 130 and 170 to 180 (Figure 4C).

The RMSD of the Cα atoms of the TrkA receptor in complex with PHUB000399 (Figure 4C) was reasonably stable, with fewer deviations and an average of approximately 3.0 Å. Larger deviations in the RMSD of PHUB000399 atoms were observed during the initial 25 ns simulation period; thereafter, the RMSD stabilized, with an average of approximately 2.0 Å. The results suggest that PHUB000399 stabilized the conformation of the TrkA receptor, as indicated by fewer deviations in the RMSD of Cα atoms. Although a gradual increase was observed in the RMSD pattern, the complex equilibrated and stabilized at 3.0 Å, with no significant perturbation after 22 ns. This indicates that an equilibrium state was achieved earlier in the simulation and that this complex followed a stable pathway during the simulation. In addition, fluctuations in residues in the loop region encompassing residues 100 to 130 were lower than those of complexes with the co-crystal ligand and Entrectinib. The overall RMSF (Figure 4C) of TrkA residues in complex with PHUB000399 was lower and below 2.4 Å, except for residues 170 to 180, which belong to the loop region.

The RMSD of the Cα atoms of the TrkA receptor in complex with NPACT01417 was significantly higher, with major deviations during the 40 to 60 ns and 90 to 100 ns simulation periods (Figure 4C). TrkA RMSD stabilized around 2 to 3.5 Å, with major perturbations at 35 to 40 ns, suggesting minimal conformational adjustments. However, ligand RMSD fluctuated between 1.0 and 4.5 Å, reflecting its dynamic behavior within the binding site. These fluctuations may indicate that the ligand explored different conformations. The relatively stable protein RMSD, along with ligand adaptability, suggests a well-formed and robust binding pocket. The RMSF of NPACT01417 atoms was also significantly higher, corroborating the higher RMSD of the Cα atoms of the TrkA receptor (Figure 4C). Larger fluctuations were observed in residues belonging to naturally dynamic loop regions encompassing residues 10 to 75 and 100 to 130. Green bars below the RMSF curve correlate with residues involved in interactions, emphasizing their structural stability and importance in the binding process.

**Figure 5.**
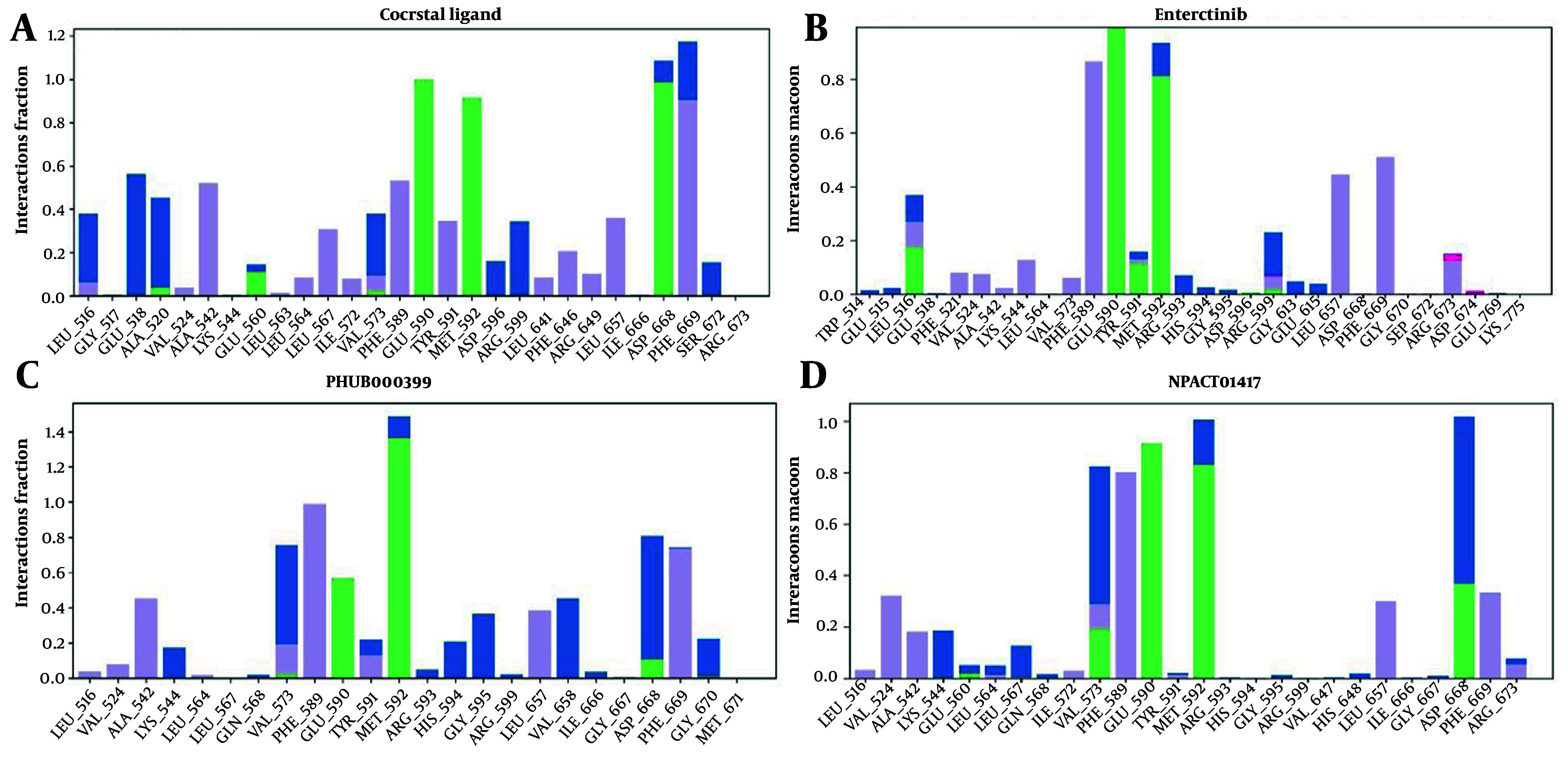
TrkA kinase-co-crystal ligand contact interactions, TrkA receptor-Entrectinib contact interactions, TrkA receptor-PHUB000399 contact interactions, and TrkA receptor-NPACT01417 contact interactions.

The co-crystal ligand formed key hydrogen bonds with Glu590, Met592, and Asp668. A few conformations also established hydrogen bonds with Ala520, Glu560, and Val573. Ala542, Leu567, Phe589, Tyr591, Leu657, and Phe669 exhibited hydrophobic interactions, such as π-π or π-alkyl interactions. Nitrogen atoms from the benzopyrrole ring and the oxygen atom from the urea component of the co-crystal ligand were mainly involved in hydrogen-bond formation. In addition, bond fraction analysis revealed a stable half-life for each interaction during the simulation. For example, the water-mediated interaction between the ligand and Glu518 was sustained in 32% of the whole trajectory; similarly, Ala520 sustained an interaction in only 41% of the trajectory. Other key residues, such as Glu590, showed a half-life of 99% in the simulation trajectory, whereas Met592 showed 92% during the simulation. Furthermore, Arg599, which established a water-mediated interaction, was sustained in only 34% of the trajectory, whereas Asp668 showed a direct interaction with a half-life of 98%. Finally, Phe669 showed a stable interaction in 64% of the whole trajectory. The interaction pattern and fraction analysis are shown in Figure 5. Overall, these analyses show that ligand binding causes structural destabilization, which produces an inhibitory cascade that inhibits the role of TrkA protein in various diseases. Destabilization refers to disruption of the active conformation of the TrkA kinase domain, specifically the DFG-in motif and αC-helix orientation. By preventing the protein from maintaining the active, stable conformation required for ATP catalysis, the ligand effectively halts the downstream signaling cascade.

Entrectinib formed key hydrogen bonds with Glu590 and Met592. Both hydrogen bonds were formed with nitrogen atoms of the benzopyrrole ring of Entrectinib (Figure 5A). Occasional hydrogen bonds were observed with Leu516 and Tyr591. Phe589, Leu657, and Phe669 formed hydrophobic interactions. Dashed lines represent hydrogen bonds, indicating strong interactions with residues such as Phe589, Glu590, and Met592. The percentages shown in Figure 5 indicate occupancy or stability during the simulation. For example, Glu590 showed 93% occupancy, whereas Met592 showed a 70% interaction half-life with the ligand. The hydrophobic interaction involving Phe669 was present in 59% of the total trajectory. All these interactions are essential for stabilizing the ligand position inside the binding pocket. The interaction fractions for Phe589, Glu590, and Met592 were highest, suggesting that these residues are key contributors to ligand binding. Bars are color-coded to differentiate interaction types, such as hydrogen bonds, hydrophobic contacts, and water-bridged interactions. The dominance of 1 color over others provides insights into the nature of binding.

PHUB000399 was stabilized at the binding site through hydrogen-bond interactions with Glu590 and Met592 and hydrophobic interactions with Ala542, Phe589, Leu657, and Phe669. The phenolic hydroxyl groups were involved in key hydrogen bonds with Glu590 and Met592. Phe589 and Phe669 formed π-π stacking interactions with the aromatic benzene ring of chromone-4-one and the phenyl ring at the second position of the chromen-4-one ring, respectively. The hydroxyl group at the seventh position established a water bridge through Val573, and the carbonyl oxygen at the fourth position established a water bridge through Asp668. Various contacts were established between the ligand and receptor, including hydrogen bonds. Asp668 established a bond with the ligand oxygen atom, with 40% occupancy. Glu590 showed a hydrogen-bonding interaction with the hydroxyl oxygen of the ligand, with a half-life of 56%. Met592 engaged with the ligand in hydrogen bonds, with occupancies of 62% and 68%. Val658 maintained hydrogen bonding through water mediation (H_2O), with 36% occupancy. Val573 and the oxygen atoms of the ligand each showed 40% occupancy, and Phe589 showed major hydrophobic interactions with the ligand, with 89% occupancy during the simulation. Phe669 showed contact with the ligand, with 37% hydrophobic occupancy. Thus, these hydrophobic contacts were additionally supported by the aromaticity of the ligand and by hydroxyl (-OH) groups that served as mediators of these interactions. The hydroxyl groups were responsible for hydrogen bonding, whereas the aromatic regions generated strong hydrophobic contacts. The percentages indicate how often these interactions occurred during the simulation. Higher occupancy indicates stronger and more stable interactions, whereas lower percentages reflect more transient contacts (Figure 5). These favorable interactions may have stabilized the conformation of PHUB000399 at the binding site.

**Figure 6.**
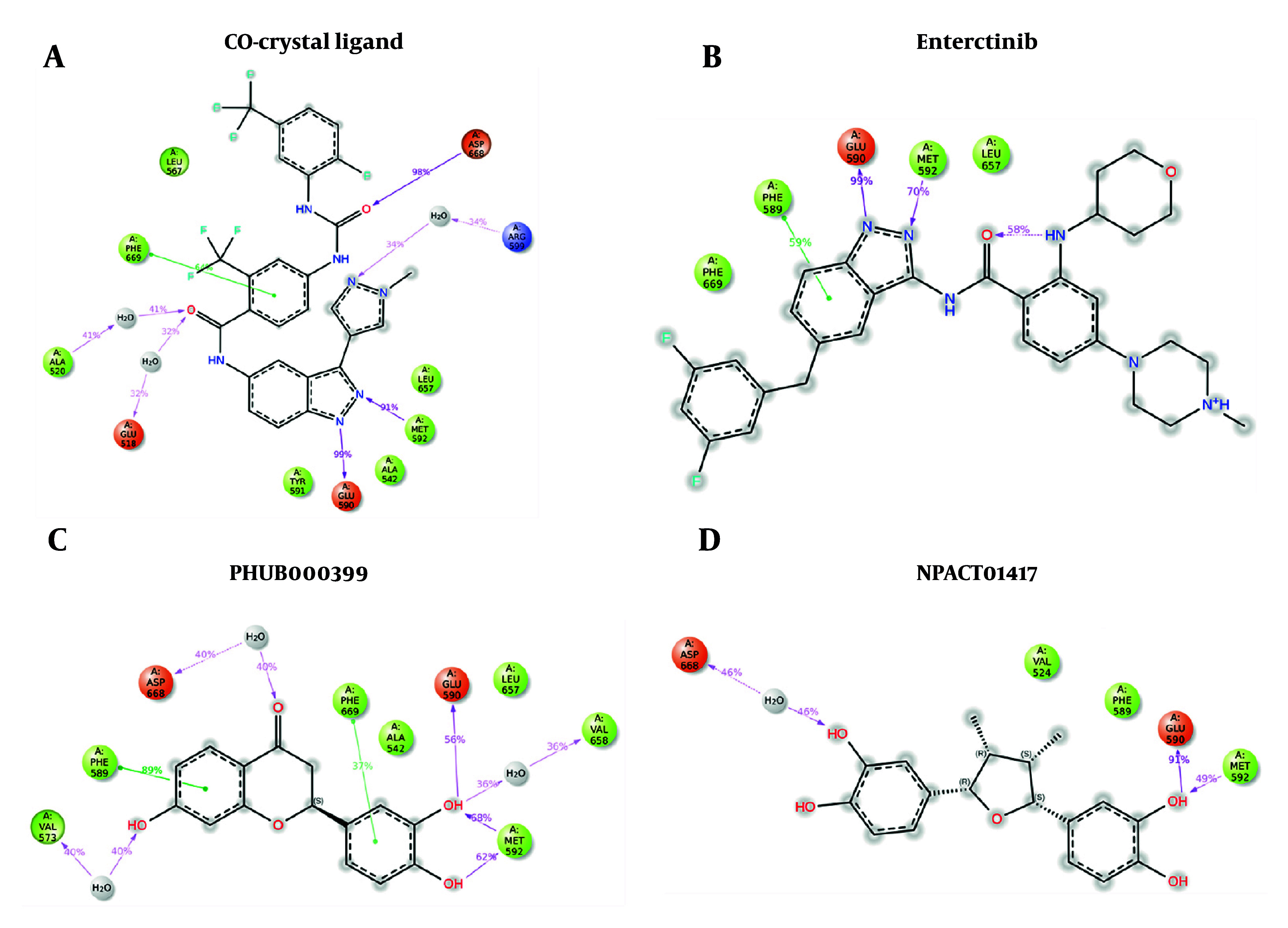
Ligand interaction diagram.

Figure 5 describes the interactions between the ligand and TrkA active-site residues in detail. Different types of interactions are shown, including hydrogen bonding represented by purple dashed lines and hydrophobic interactions represented by green dashed lines, each annotated with a percentage representing the occupancy or stability of the interaction throughout the simulation. For example, hydrogen-bond interactions were established with Asp668 and Glu590, which formed 46% and 91% occupancy with the ligand, respectively. These percentages indicate the frequency with which these bonds were maintained over the trajectory. Hydrophobic interactions are illustrated by residues such as Phe589, which had constant interactions with an occupancy of 95%. These strong and consistent interactions tethered the ligand firmly into the binding pocket, with additional stabilizing effects from water-mediated hydrogen bonds, such as those involving Val524. The multiple hydroxyl groups and aromatic rings of these ligands facilitate both polar and non-polar interactions, which are critical for binding affinity and specificity. Figure 6 presents a histogram showing the fraction of total time during which specific residues maintained interactions with the ligand. The colors of each bar represent different types of interactions: purple for hydrogen bonds, green for hydrophobic contacts, and blue for water-mediated contacts. Residues such as Glu590, Phe589, and Met592 showed the highest interactions, emphasizing their importance in ligand binding. The dominance of certain interaction types for specific residues, such as hydrogen bonding for Glu590 and hydrophobic interaction for Phe589, indicates the diverse mechanisms through which ligand binding is stabilized in the binding pocket. NPACT01417 formed hydrogen bonds with Glu590, Met592, and Asp668. Val524, Phe589, Leu657, and Phe669 formed hydrophobic interactions. The phenolic hydroxyl groups established key hydrogen-bond interactions. Protein-ligand contact interactions are shown in Figure 5.

The ligand interaction diagram is presented in Figures 6. Detailed protein-ligand contact interactions and ligand interaction diagrams for all complexes are presented in Figures 5 and 6, respectively.

### 4.3. MM-GBSA Binding Free Energy Calculations

The MM-GBSA binding free energy calculations revealed a hierarchy of binding affinities among the tested ligands, with the co-crystal ligand showing a strong interaction with TrkA kinase (-84.14 ± 5.22 kcal/mol). This result is expected because co-crystal ligands are often tailored for optimal binding to their target protein, leveraging a highly complementary fit within the binding pocket. The high binding affinity likely arises from a balance of hydrophobic interactions, hydrogen bonding, and favorable electrostatic contributions that stabilize the complex. This serves as a benchmark for assessing the performance of other ligands. The standard inhibitor Entrectinib exhibited a lower binding affinity of -73.03 ± 6.17 kcal/mol compared with the co-crystal ligand. Although still strong, this suggests that Entrectinib may not achieve the same level of interaction efficiency or complementarity within the binding pocket. Nonetheless, its binding strength underscores its viability as a therapeutic inhibitor of TrkA kinase, given its ability to effectively engage key residues.

**Figure 7.**
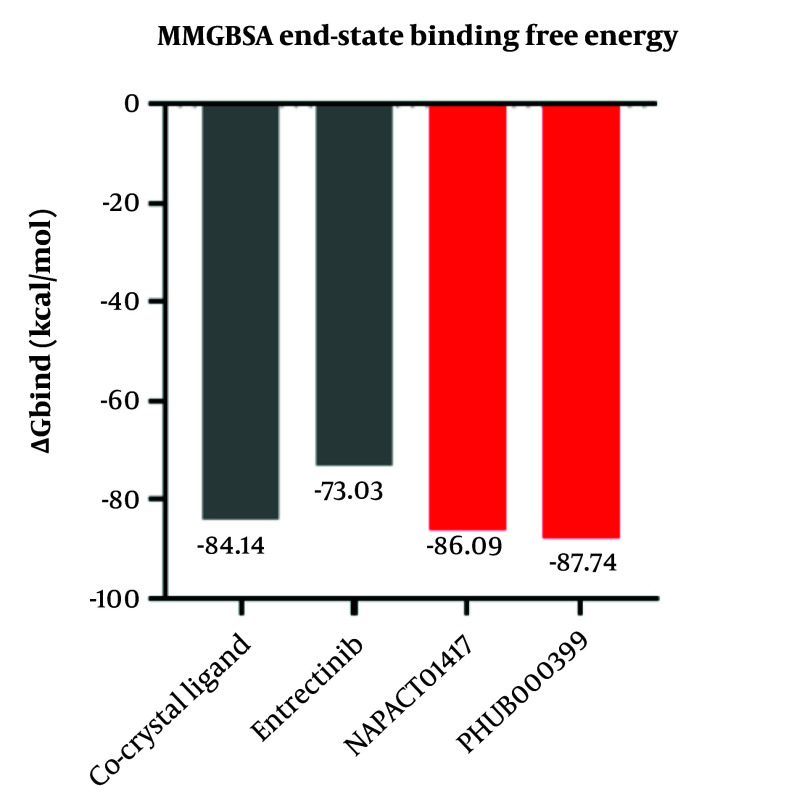
End-state MM-GBSA binding free energy calculations on molecular dynamics trajectories of TrkA kinase with ligands, including Entrectinib, the co-crystal ligand, NPACT01417, and PHUB000399.

Interestingly, the test ligands NPACT01417 and PHUB000399 demonstrated binding affinities of -86.09 ± 4.89 and -87.74 kcal/mol, respectively, which were higher than those of the co-crystal ligand and Entrectinib (Figure 7). This suggests that NPACT01417 and PHUB000399 may have a stronger binding mechanism and could potentially serve as alternatives to Entrectinib, particularly if other factors, such as bioavailability or toxicity profiles, are more favorable. Their binding strength highlights their capacity to interact robustly with TrkA kinase, likely through hydrophobic and van der Waals forces. A visual comparison of the MM-GBSA binding free energies for all ligands is provided in Figure 7.

Across all complexes, hydrophobic interactions emerged as the dominant stabilizing force. This is consistent with the nature of kinase-ligand interactions, in which hydrophobic regions of the binding pocket play a critical role in driving affinity. These results highlight the potential of the test ligands, particularly NPACT01417 and PHUB000399, as promising candidates for further development, with opportunities to optimize their interactions to match or exceed those of standard inhibitors. It has been noted that several compounds identified through this virtual screening, such as a flavonoid derivative, have previously documented biological and pharmacological effects (37).

### 4.4. Essential Dynamics of the TrkA-Ligand Complexes

A cross-correlation comparison was performed using the dynamic cross-correlation matrix approach to show the dynamics of the unbound system for each complex, including the control, Entrectinib, PHUB000399, and NPACT01417 complexes. Positive correlations are shown in blue, whereas negative correlations are shown in red. The red color, or negative correlations, across the protein indicates flexible interactions and widespread residue communication. In contrast, Entrectinib binding produced a more structured map with more pronounced and defined clusters of correlations, indicating stabilization of certain regions due to the presence of the ligand. Furthermore, the PHUB000399-TrkA complex showed a slightly more dispersed correlation pattern than Entrectinib, although it still showed a stabilized pattern compared with the unbound state. This indicates differences in how each ligand influences residue interactions, with Entrectinib potentially inducing more localized effects than PHUB000399. Similarly, NPACT01417-TrkA also demonstrated negative correlations across the protein, indicating flexible interactions and widespread residue communication. The dynamic cross-correlation matrix results for each complex are provided in Figure S1 in the Supplementary File.

The free energy landscape was constructed using the principal components from each complex. For the co-crystal ligand, a clear and moderately deep basin in the free energy landscape suggested a system with well-defined low-energy conformations but some flexibility. When Entrectinib bound, the energy landscape became unusually steep, with higher energy up to approximately 14 kcal/mol, indicating a more constrained conformational space. Deeper and broader low-energy basins were observed, suggesting lower flexibility and multiple stable conformational states. The PHUB000399 complex represented an intermediate case, with slightly lower overall energy, approximately 9 kcal/mol, than the Entrectinib complex. The energy basins were distinct but appeared to allow more flexibility than the Entrectinib complex, indicating that PHUB000399 stabilizes the complex without excessively constraining the system. Similarly, the TrkA-NPACT01417 complex showed behavior similar to that of the co-crystal control, defining a single and moderately deep state during the simulation. This demonstrates the differential effects of ligand binding on the dynamic behavior of TrkA. The PCA-based free energy landscape results for each complex are provided in Figure S2 in the Supplementary File.

The free energy landscape as a function of the radius of gyration and RMSD was calculated for each complex, with color-coded blue and red regions representing lower and higher energy states, respectively. The blue regions indicate thermodynamically favorable states during simulation. For example, the 3-dimensional free energy landscape of the co-crystal control was dominated by moderate valleys of reasonable and low free energy basins, accommodating a stable system able to sample multiple conformational states. Entrectinib binding resulted in narrower valleys with higher free energy, up to approximately 5 kcal/mol, indicating a more compact and rigid structure with limited conformational freedom. In contrast, the PHUB000399 complex exhibited broader valleys than Entrectinib, suggesting some stabilization without substantial conformational restriction. For the 7XBI-NPACT01417 complex, the graph indicates that, in the presence of the ligand, the protein stabilizes into specific conformational states represented by well-defined deep basins. These stable states reflect structural adjustments that the protein undergoes to accommodate the ligand. The ligand likely shifts the energy landscape by stabilizing certain conformations or introducing new metastable states, represented by shallow basins, that may reflect intermediate structures important for the functional mechanisms of the protein. Conversely, the free energy landscape of the co-crystal control ligand serves as a baseline, with Entrectinib imposing even greater rigidity than the co-crystal ligand, whereas PHUB000399 occupies a position between flexibility and stability. The free energy landscape results for each complex are provided in Figure S3 in the Supplementary File.

### 4.5. Conclusions

This research successfully identified NPACT01417 and PHUB000399 as promising candidates with significant inhibitory potential against TrkA for cancer therapy. The structure-based drug design approach, which included molecular docking, molecular dynamics simulations, and energy calculations, yielded valuable insights into the behavior, stability, and energy profiles of the ligand-protein complexes. These findings may facilitate the development of novel TrkA inhibitors, positioning NPACT01417 and PHUB000399 as lead compounds in this study. However, further in vivo and in vitro experiments are necessary to validate these molecules as viable anti-cancer therapies. Additionally, in vitro kinase assays and cell-based validation are essential to confirm their efficacy against cancer cell migration and resistance.

ijpr-25-1-166946-s001.pdf

## Data Availability

The dataset presented in this study is available on request from the corresponding author during submission or after its publication. The data are not publicly available due to restrictions related to confidential laboratory records and proprietary analysis workflows.
