# Supplementary material for: In silico Identification of Natural Compounds as Potential TrkA Inhibitors for Anticancer Drug Development
Source: Iran J Pharm Res. 2026 May 25;25(1):e166946. doi: 10.5812/ijpr-166946 (PMC13389379; doi:10.5812/ijpr-166946)

Figure S1. Dynamic cross-correlation analysis (DCCM) results. **A** TrkA-co-crystal ligand comp lex B) TrkA-Entrectinib complex, **C** TrkA-PHUB000399 complex and **D** TrkA-NPACT01417 complex.

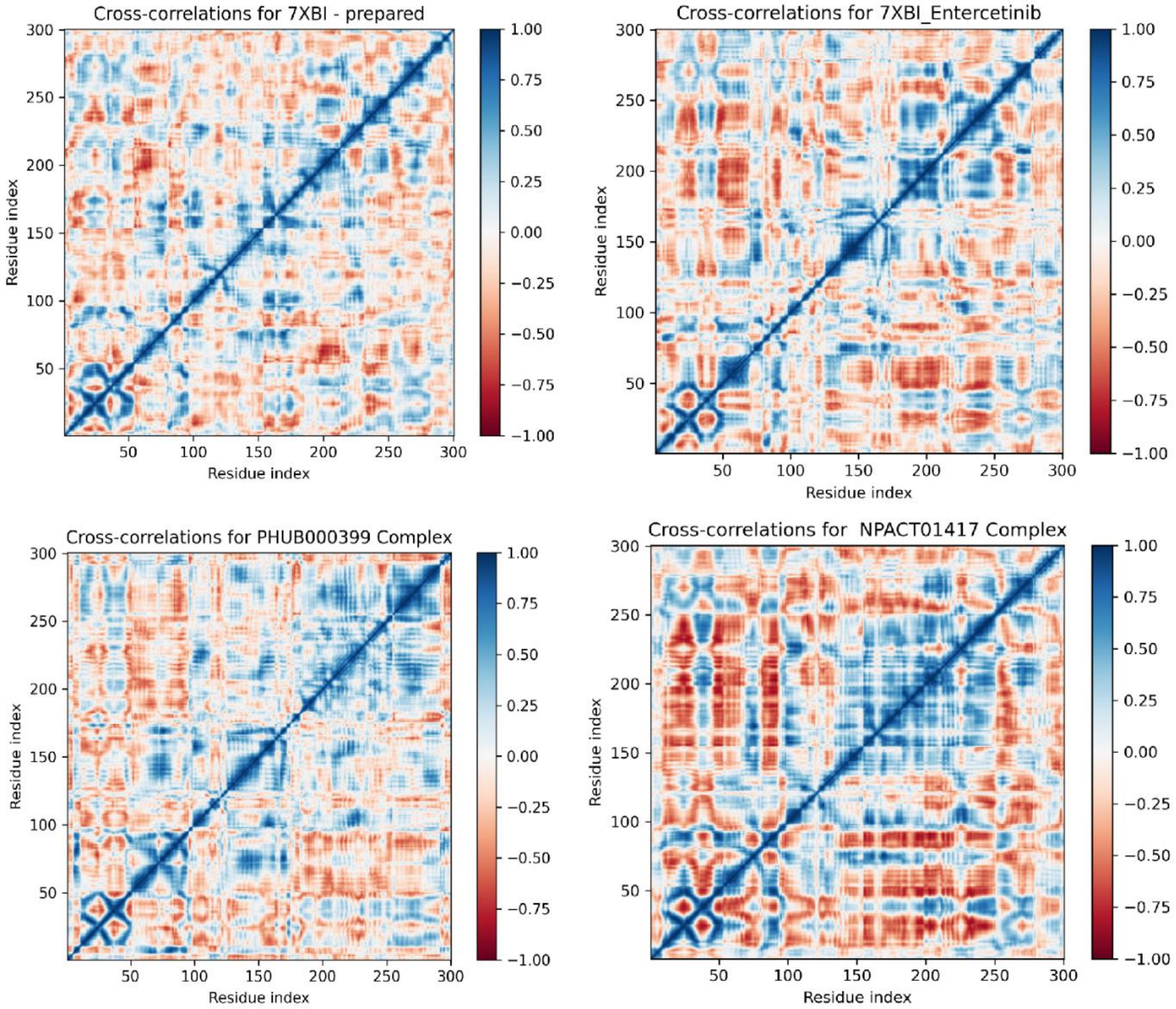

Figure S2. PCA analysis results. **A** TrkA-co-crystal ligand (Control) complex, **B** TrkA-Entrectinib complex, **C** TrkA-PHUB000399 complex and D) TrkA-NPACT01417 complex.

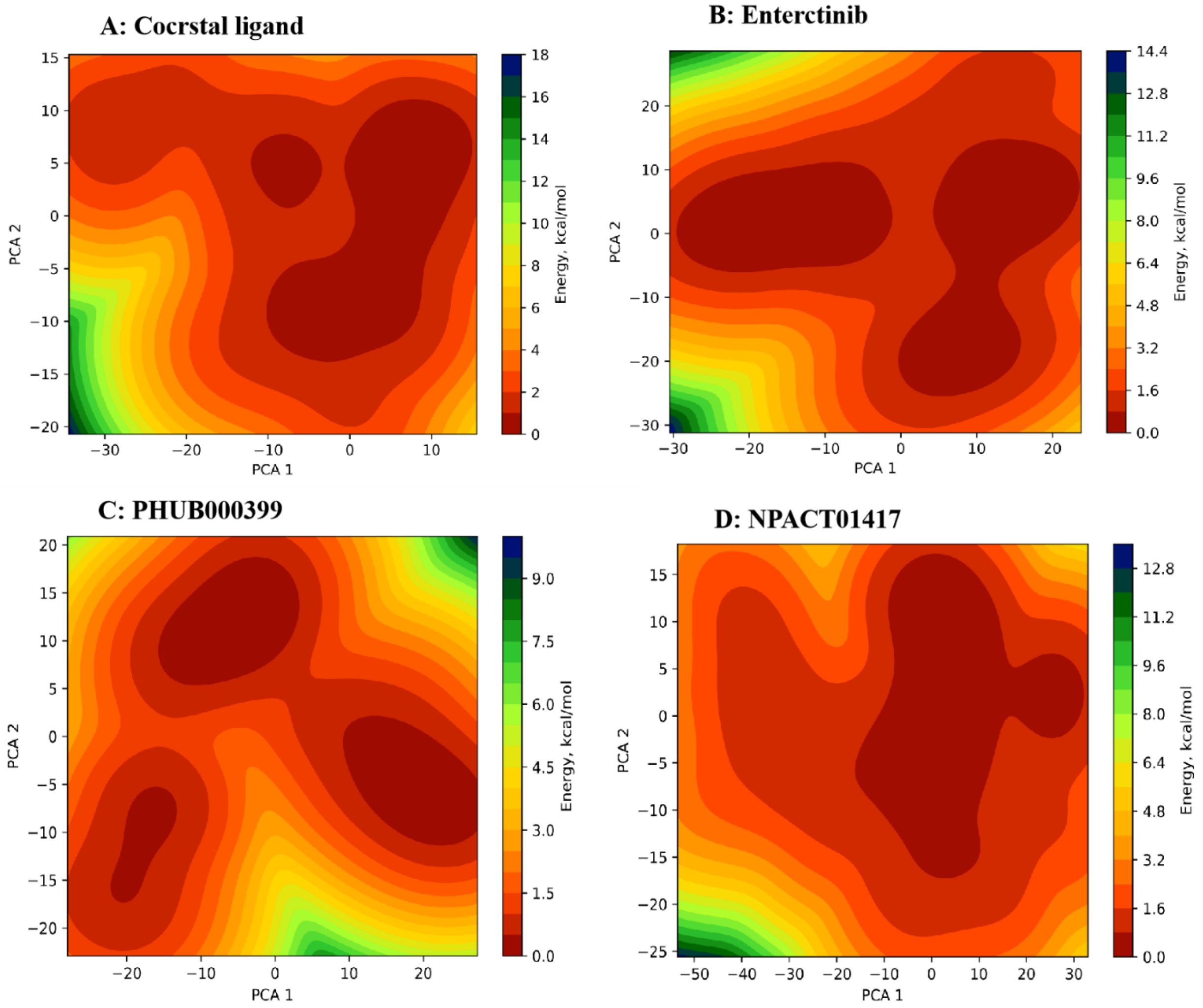

Figure S3. FEL analysis results. **A** TrkA-co-crystal ligand complex, **B** TrkA-Entrectinib compl ex, **C** TrkA-PHUB000399 complex and **D** TrkA-NPACT01417 complex.

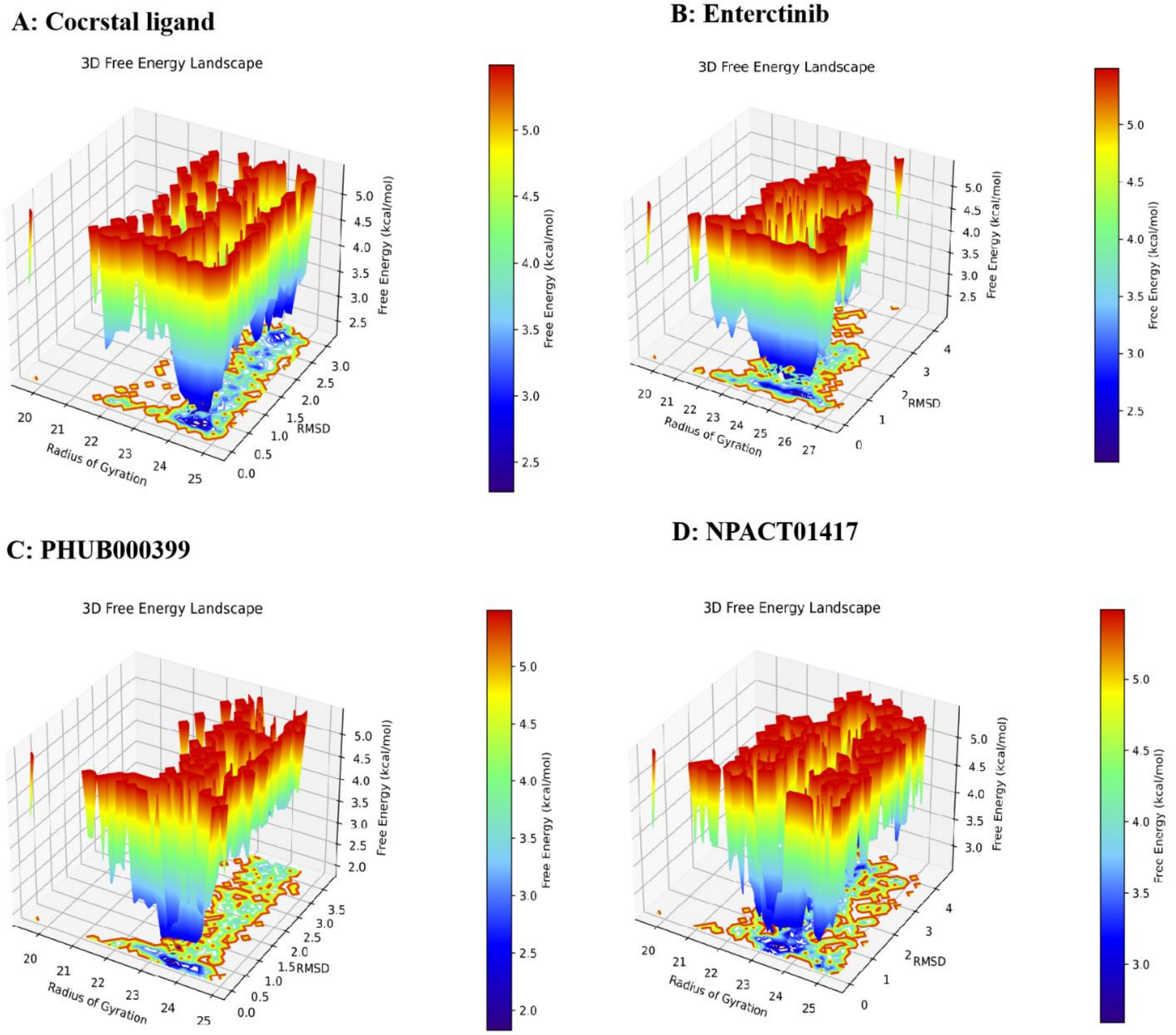

Supplement: ijpr-25-1-166946-s001.pdf [file ijpr-25-1-166946-s001.pdf]
